# Structural–functional coupling abnormalities in temporal lobe epilepsy

**DOI:** 10.3389/fnins.2023.1272514

**Published:** 2023-10-19

**Authors:** Xiaoting Huang, Yangsa Du, Danni Guo, Fangfang Xie, Chunyao Zhou

**Affiliations:** ^1^Department of Neurology, Xiangya Hospital, Central South University, Changsha, China; ^2^Department of Radiology, Xiangya Hospital, Central South University, Changsha, China; ^3^Department of Neurosurgery, Xiangya Hospital, Central South University, Changsha, China

**Keywords:** functional connectivity, structural connectivity, temporal lobe epilepsy, structural–functional coupling, human brain

## Abstract

**Background:**

Nowadays, researchers are using advanced multimodal neuroimaging techniques to construct the brain network connectome to elucidate the complex relationship among the networks of brain functions and structure. The objective of this study was to evaluate the coupling of structural connectivity (SC) and functional connectivity (FC) in the entire brain of healthy controls (HCs), and to investigate modifications in SC–FC coupling in individuals suffering from temporal lobe epilepsy (TLE).

**Methods:**

We evaluated 65 patients with TLE matched for age and gender with 48 healthy controls. The SC–FC coupling between regions was determined, based on which whole-brain nodes were clustered. Differences in the coupling among the three groups of nodes were compared. To further validate the results obtained, the within-cluster coupling indices of the three groups were compared to determine the inter-group differences.

**Results:**

Nodes were divided into five clusters. Cluster 1 was primarily located in the limbic system (*n* = 9/27), whereas cluster 5 was mainly within the visual network (*n* = 12/29). By comparing average cluster SC–FC coupling in each cluster of the three groups, we identified marked discrepancies within the three cohorts in Cluster 3 (*p* = 0.001), Cluster 4 (*p* < 0.001), and Cluster 5 (*p* < 0.001). Post-hoc analysis revealed that the SC–FC coupling strengths in LTLE and RTLE were significantly lower than that in HCs in Cluster 3 (PL = 0.001/PR = 0.003), Cluster 4 (PL = 0.001/PR < 0.001), and Cluster 5 (PL < 0.001/PR < 0.001). We also observed that the within-cluster SC–FC coupling in cluster 5 of left- and right TLE was significantly lower than in HCs (PL = 0.0001, PR = 0.0005).

**Conclusion:**

The SC and FC are inconsistently coupled across the brain with spatial heterogeneity. In the fifth cluster with the highest degree of coupling in HCs, the average SC–FC coupling index of individuals with TLE was notably less than that of HCs, manifesting that brain regions with high coupling may be more delicate and prone to pathological disruption.

## 1. Introduction

The relationship between the structural and functional of the neural network system continues to be studied and explored in neuroscience, to decipher how the organization of the neural connectivity network impacts its functionality. The functional connections are constructed by structural connections throughout the entire brain, resulting in different networks of brain functions. Researchers are using advanced multimodal neuroimaging techniques, including diffusion tensor imaging (DTI) and resting state functional magnetic resonance imaging (rs-fMRI) to construct the brain network connectome to elucidate the complex relationship among the networks of brain functions and structure (Kelly et al., 2012; Smith et al., 2013). The correlation between the functional and structural connection matrix at the subject level is found, and the SC–FC coupling is allowed to be quantified (Skudlarski et al., 2008). Many studies show that the construction of SC and FC (Skudlarski et al., 2008; Honey et al., 2009) are correlated in the brain network of healthy controls (HCs), and a positive correspondence between SC and FC has also been described in certain neural circuits, particularly in the visual network (van den Heuvel et al., 2009), sensorimotor system (Koch et al., 2002), and default mode network (DMN) (Raichle et al., 2001; Greicius et al., 2003).

It is noteworthy that temporal lobe epilepsy (TLE), which is characterized by epileptogenic foci, displays distinct alterations in the SC–FC coupling. TLE is a prevalent form of focal epilepsy. However, studies have shown that, in addition to abnormal connectivity at the lesion site, there are also significant abnormalities in other extensive brain networks, mainly involving the extratemporal structures and bilateral temporal lobes (Engel et al., 2013). These findings have resulted in the concept that TLE has been a “network disease” (Bonilha et al., 2012; Richardson, 2012; Bassett and Sporns, 2017). Several studies have demonstrated an interruption of the functional and structural network in TLE (Chiang et al., 2015; Bernhardt et al., 2016; Trimmel et al., 2021), which is manifested as a global or regional decrease in connectivity strength (Voets et al., 2012). Generally speaking, the change in TLE is mainly characterized by an extensive decrease in FC of the whole brain, as shown by DMN (Voets et al., 2012) and the subcortical network (Li et al., 2022), while the alterations in the SC are relatively limited, and generally confined to the temporal lobe and adjacent areas (Liu et al., 2016; Galovic et al., 2019; Foit et al., 2021). However, because the change patterns of FC and SC differ considerably in TLE, limited research has investigated the association between the lateralization of TLE and SC–FC coupling using DTI and rs-fMRI; hence, it is difficult to describe these differences quantitatively. Previous studies indicate significant spatial heterogeneity in the coupling between SC and FC throughout the entire brain, allowing for the classification of the whole-brain network based on the coupling effects observed in local brain regions. Hence, we hypothesize that utilizing clustering algorithms can effectively sort the local brain regions into clusters with varying degrees of coupling, leading to a comprehensive analysis of SC–FC coupling characteristics in patients with TLE.

The objective of this study was to examine the characteristics of SC–FC coupling in TLE on the network and modular scales. To achieve this objective, we retrospectively reviewed 65 patients with drug-resistant mesial TLE. We proposed to (1) map the SC–FC coupling characteristics in HCs and determine how they are distributed and enriched at different pre-identified functional modules, and (2) determine alterations in the SC–FC coupling of patients with TLE at different modules.

## 2. Materials and methods

### 2.1. Participants

This study involved a cohort of 65 individuals diagnosed with drug-resistant unilateral TLE. Among them, 34 had left-sided TLE, while 31 had right-sided TLE. We determine the laterality of a patient’s lesion based on a combination of their semeiotic findings, imaging findings, and EEG analysis. Forty-eight matched individuals with no prior record of epilepsy or any other long-term neurological disease or mental illness were included as HCs (see Table 1 for details). Patients with TLE were enrolled from the Department of Functional Neurosurgery at Xiangya Hospital within the period of 2018 to 2022. Drug-resistant TLE was identified in accordance with the diagnostic criteria established by the International League Against Epilepsy (Fisher et al., 2017). The inclusion criteria were (1) a confirmed diagnosis of drug-resistant epilepsy and (2) the absence of contraindications for surgical resection. Conversely, the exclusion criteria comprised pre-operative intracranial monitoring, progressive neurologic disorder, localized abnormality situated beyond the temporal region, and the coexistence of epilepsy with severe mental disorders.

**Table 1:** Clinical and demographic data of participants.

	HC	LTLE	RTLE	*p-*value
No. of participants, *n*	48	34	31	/
Age, years	30.3 ± 8.4	29.9 ± 8.9	30.6 ± 11.7	*p* = 0.951
Years of education	11.6 ± 2.6	10.6 ± 3.5	10.7 ± 3.2	*p* = 0.287
Sex (M/F)	30/18	17/17	17/14	*χ*^2^ = 1.322, *p* = 0.516
Handedness (L/R/A)	0/48/0	0/34/0	0/31/0	/
Age at seizure onset, years (mean ± SD)	/	15.8 ± 10.6	16.5 ± 14.5	*p* = 0.958
Years of seizure duration, (mean ± SD)	/	14.9 ± 10.0	14.6 ± 9.4	*p* = 0.913
Presence of HS, *n*	/	25	26	/
Information of medication (mean ± SD)	/	2.2 ± 0.5	2.3 ± 0.6	*p* = 0.553

HC, healthy control; LTLE, left temporary lope epilepsy; RLE, right temporary lope epilepsy; (L/R/A), (left/right/ambidextrous); HS, hippocampal sclerosis; TLE, temporary lope epilepsy.

The present study obtained approval from both the Ethics Committee and Institutional Review Board of Xiangya Hospital. All subjects provided written informed consent in compliance with the principles outlined in the Helsinki Declaration.

### 2.2. MRI acquisition

The rs-fMRI data for all participants were obtained utilizing a 3.0 Tesla Siemens Prisma MRI system equipped with a standard 32-channel head coil. rs-fMRI images were collected using echo plane imaging (EPI) sequences. The specific settings were as listed below: TR = 720 ms, TE = 37 ms, 64 axial slices with 2.5 mm thickness and 2.5 mm gap, flip angle = 52°, matrix size = 90 × 90, field of view (FOV) = 225 mm × 255 mm, and voxel size = 2.5 mm × 2.5 mm × 2.5 mm. Each resting-state scan had a duration of 9.456 min, giving rise to 788 volumes.

DTI data were obtained using a multi-shell EPI sequence with the subsequent specifications: TE = 72 ms, TR = 5,400 ms, resolution = 2.0 × 2.0 × 2.0 mm, flip angle = 90°, axial slices = 75, voxel size = 1.6 *×* 1.6 *×* 1.6 mm, FOV = 215 *×* 215 mm; b = 0/1000/2000/3000 s/mm^2^, number of directions = 96, EPI factor = 154.

### 2.3. Functional connectivity network construction

The rs-fMRI data were preprocessed using the GRETNA (Wang J. et al., 2015) toolbox (GRETNA; https://github.com/sandywang/GRETNA), based on SPM 12.^1^ The preprocessing procedures are briefly detailed here. (1) The data was converted from Digital Imaging and Communications in Medicine (DICOM) format to Neuroimaging Informatics Technology Initiative (NIfTI) format. (2) The initial 18 time-point volumes were excluded to ensure patients had sufficient time to reach a resting state and prevent magnetization saturation. This resulted in a total of 770 volumes. (3) Slice timing was corrected and (4) spatial realignment: motion correction was achieved by applying a linear registration method using a spatial realignment algorithm with 12 degrees of freedom. (5) The EPI template was applied for standardization to the Montreal Neurological Institute (MNI) space. The algorithms used for standardization is called normalization, which is fully developed by SPM. It is basically a combination of geometric and intensity-based methods to align the data, instead of linear affine transformation. Specifically, it uses a distortion model based on the epipolar geometry constraints to align the data from different subjects, and then applies a nonlinear transformation to register the data to a reference space. (6) Spatial smoothing was performed. The final steps were (7) temporally detrending and (8) regressing out the global signal.

The Pearson correlation between the average time series of each region pair in the Brainnetome (BN) atlas was used to calculate the FC matrix (Fan et al., 2016) resulting in a 246 × 246 FC matrix for each patient. The FC matrix was then subjected to Fisher’s *Z* transformation to achieve a Gaussian distribution. A sparsity threshold of 10% was applied to the FC matrix. To quantify the within- and between-modular FC, we assigned each subregion to a specific functional network based on the Yeo-7 atlas (Yeo et al., 2011). The 7 networks are: limbic system, default network, dorsal attention network, sensorimotor network, visual networks, ventral attention network and frontoparietal network. In addition, due to Yeo’s functional atlas not incorporating subcortical structures, which have been proven to be closely related to TLE in multiple literature sources, we define subcortical network as the eighth network. All nodes and the networks to which they belong are shown in the Supplementary Table S1.

### 2.4. Structural connectivity network construction

The DTI pre-processing pipeline was applied using FSL.^2^ Diffusion data were preprocessed according to the following steps: (1) DICOM to NIfTI conversion; (2) B0 image extraction; (3) global denoising; (4) skull stripping using brain extraction tool (BET); (5) eddy current correction; and (6) estimation of basic diffusion metrics, including fractional anisotropy (FA) and mean diffusivity (MD).

To generate the white matter (WM) connectome matrix, deterministic fiber tracking was employed to trace the white matter connections between all possible pairs of nodes. The brain regions included in the BN atlas (http://www.brainnetome.org/, accessed on March 2, 2020) were selected as nodes, resulting in a total of 246 nodes. Therefore, a 246 × 246 connectome matrix was constructed for each participant and the sparsity score of the matrix was calculated to be 10.2%. The edges were weighed using inter-regional streamline counts. The SC matrix was also divided into 8 modules similar to their FC counterparts, but no sparsity threshold was set because the SCs were naturally sparse.

### 2.5. Mapping the regional SC–FC coupling index

Regional coupling was calculated by Spearman rank correlation (Baum et al., 2020), which included determining the correlation between the corresponding rows of the SC and FC matrices, but excluded the self-connection. The choice of Spearman correlation was motivated by its ease of interpretation and its suitability for non-Gaussian distributions typically observed in SC entries (Gu et al., 2021). As a result, a vector of 246 Spearman’s correlation coefficients (*r* values) was obtained, representing the regional SC–FC coupling strength.

### 2.6. *k*-means clustering analysis

Cluster analysis was conducted using MATLAB (MathWorks, Inc. California, United States). First, each node was ranked based on the average SC–FC coupling strength and the *k*-means algorithm was utilized for the whole-brain nodes of each HC. The optimal value of *k* for the clustering analysis was determined using the “elbow method.” This method involves running the *k*-means algorithm for different values of *k* and calculating the within-cluster sum of point-to-centroid distances for each partition. The goal is to identify the value of *k* at which the rate of decrease in within-cluster sums of distances starts to flatten out, resembling an “elbow” shape. This point indicates the optimum number of clusters for the data. These criteria were then combined to obtain average SC–FC coupling strength clusters of sufficient size and clinical homogeneity. Based on the above criteria, the optimal number of clusters was determined to be five (*k* = 5). The average SC–FC coupling index and the cluster coupling index of the five modules was then calculated.

### 2.7. Calculating SC–FC coupling index within each cluster

In this study, two cluster-wise SC–FC coupling algorithms were used: the first one is the cluster-averaged coupling index (ASFC) which directly measures the average coupling strength of all regions in each cluster. The second coupling algorithm is the within-cluster coupling index (CSFC). It was calculated by computing the Spearman correlation coefficient for all structural and corresponding functional connections of within the cluster.

### 2.8. Statistical analysis

All statistics were analyzed using IBM SPSS Statistics version 22 (IBM Corp., Armonk, NY, United States) that can be accessed at: https://www.ibm.com/cn-zh/spss. Based on the SC–FC coupling features, a nonparametric *t*-test was used to calculate intergroup differences among patients with left TLE (LTLE), right TLE (RTLE), and HCs. To establish the relationship between the SC–FC coupling within the fifth cluster and the year of seizure duration, partial correlation analysis were employed. Group comparisons of clinical characteristics were performed using a nonparametric *t*-test and one-way analysis of covariance (ANCOVA). In all group analyses, baseline characteristics including sex, education level, and age were set as covariates.

## 3. Results

### 3.1. Demographic and clinical characteristics

A total of 65 participants with drug-resistant TLE were retrospectively enrolled, including 34 with left TLE and 31 with right TLE. No notable variations between LTLE and RTLE patients were detected in the age of onset or seizure duration. A total of 48 age- and sex-matched HCs were part of the research sample. The three groups showed no significant distinctions in sex, age or educational level (*p* > 0.05, Table 1).

### 3.2. Five clusters in HCs

**Figure 1:**
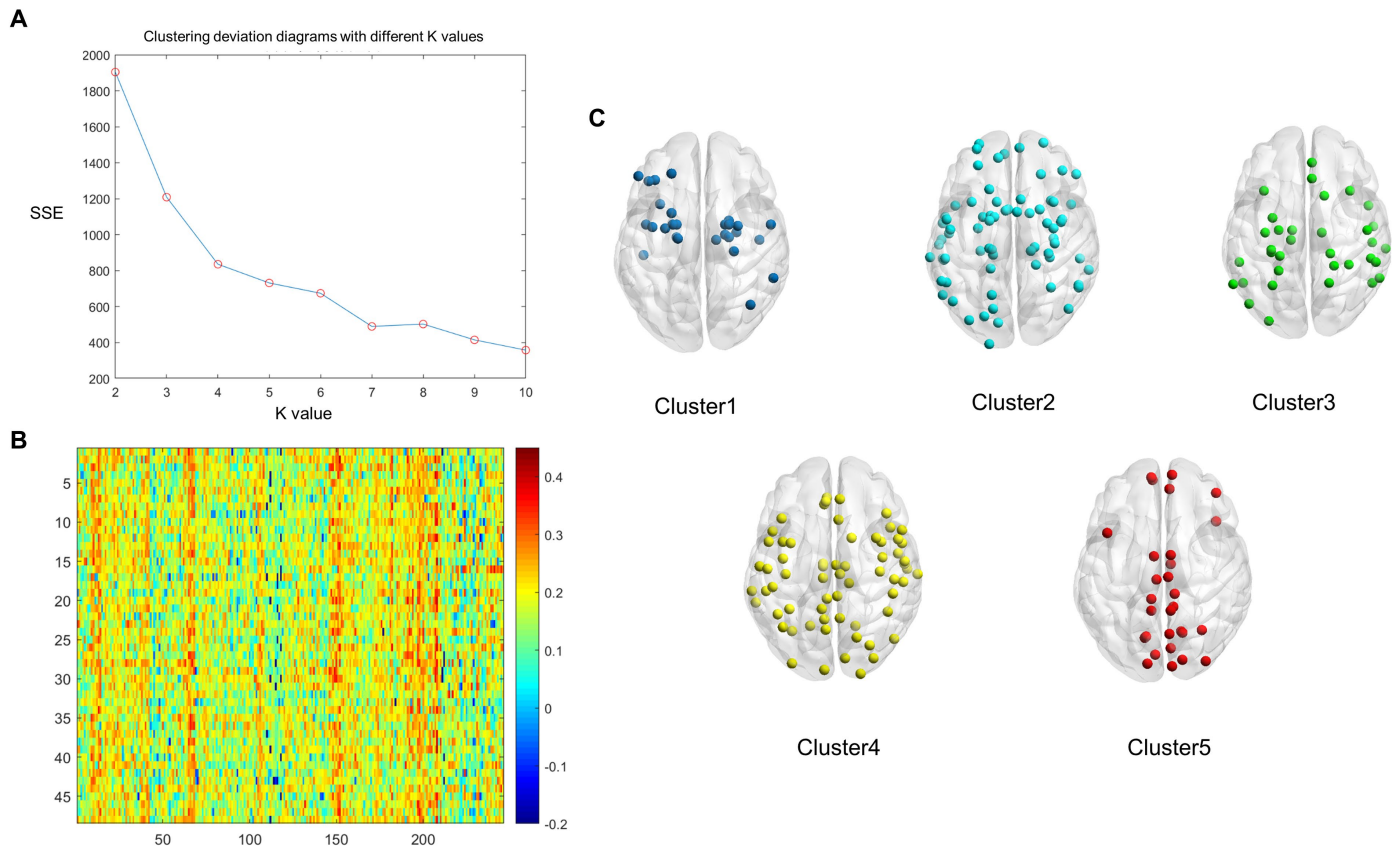
The whole-brain nodes were divided based on *k*-means cluster analysis of the average structural connectivity (SC)-functional connectivity (FC) coupling index in healthy controls (HCs). **(A)** Clustering deviation diagrams displaying various k values. **(B)** A heatmap of SC–FC coupling index of the brain network in HCs. Color: the coupling strength of structure and function. *X* axis: the nodes of the brain region; *Y* axis: every healthy individual. **(C)** A schematic diagram illustrating the distribution of each cluster node throughout the whole brain. Dark blue: Cluster 1; Light blue: Cluster 2; Green: Cluster 3, Yellow: Cluster 4, Red: Cluster 5.

**Figure 2:**
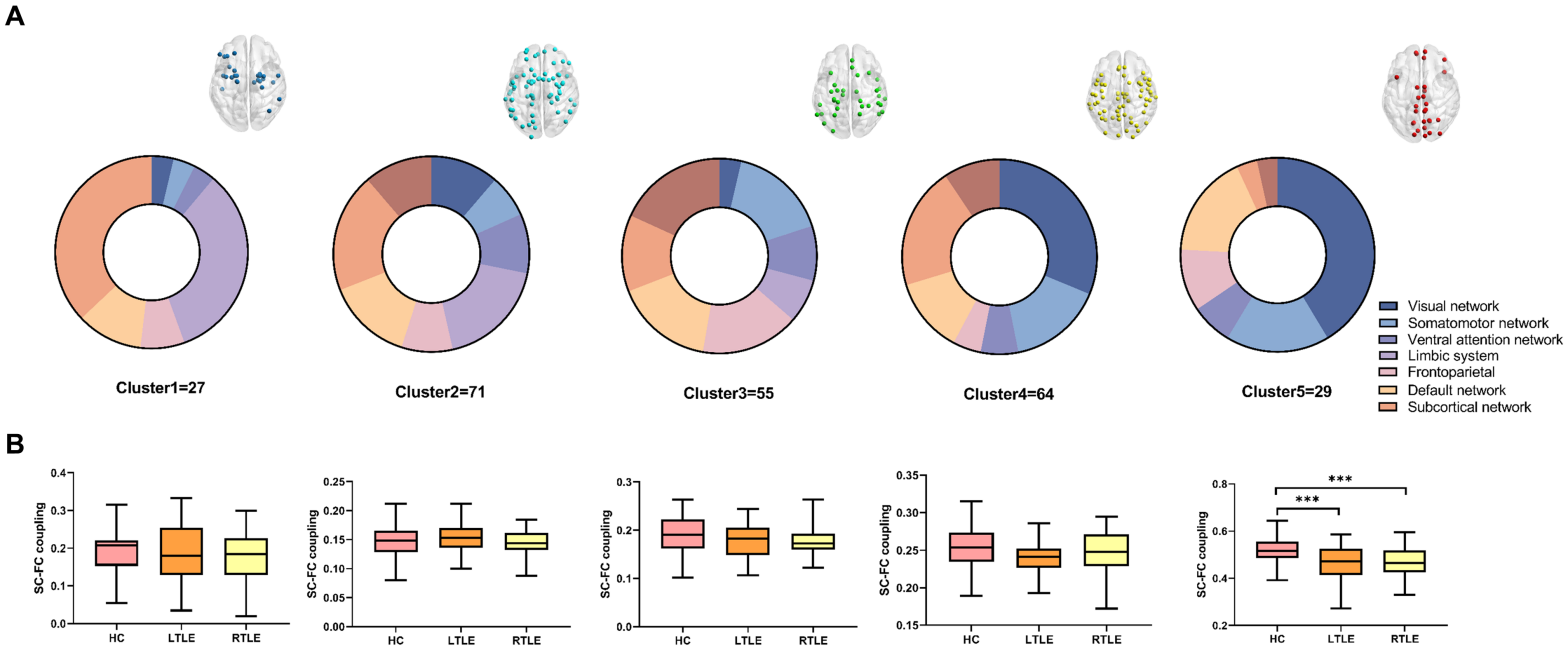
Following cluster analysis, the average structural connectivity (SC)-functional connectivity (FC) coupling index was calculated for each cluster. **(A)** Node distribution of each cluster in brain networks. **(B)** Comparison of the modular SC–FC coupling index among the three groups. The results revealed a significant decrease in the average index for the fifth cluster of temporal lobe epilepsy (TLE) compared to that in healthy controls (HCs).

We found that the optimum number of clusters was five in HCs using the elbow method. All nodes in the whole brain were then divided into five clusters by the *k*-means clustering method. The whole-brain distribution of the five resulting clusters is illustrated in Figure 1. Cluster 1 was predominantly located in the limbic system (*n* = 9/27) and the subcortical network (*n* = 10/27). Cluster 2 was in the subcortical network (*n* = 14/71), the limbic system (*n* = 13/71), and the DMN (*n* = 10/71). Cluster 3 was mainly located in the dorsal attention network (*n* = 10/55), the sensorimotor network (*n* = 9/55), the prefrontal lobe (*n* = 9/55), and the DMN (*n* = 9/55). Cluster 4 was primarily located in the visual (*n* = 20/64), subcortical (*n* = 13/64), and sensorimotor (*n* = 10/64) networks. Cluster 5, which exhibited the highest degree of coupling, was primarily located in the visual (*n* = 12/29), sensorimotor (*n* = 5/29), and the DMN (*n* = 5/29) (Figure 2A).

### 3.3. Inter-group comparison of the five clusters

**Figure 3:**
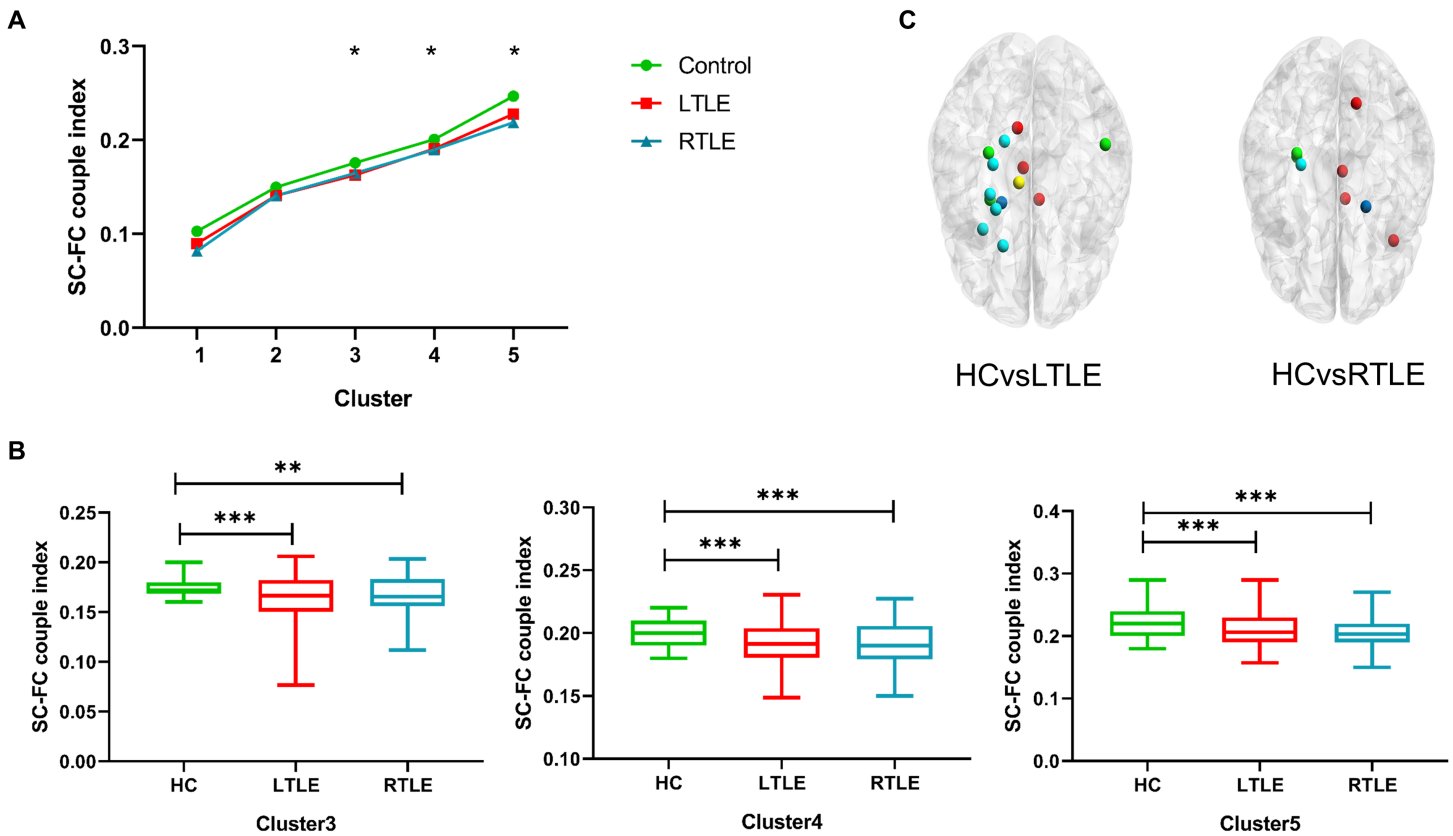
The SC–FC coupling index was calculated for each node in the brain network, and the average index was contrasted among the three groups. **(A)** Line chart indicating significant discrepancies (marked with *) in Clusters 3–5 among the three groups. **(B)** Differences in Clusters 3–5 were found between left temporal lobe epilepsy (LTLE), right temporal lobe epilepsy (RTLE), and healthy controls (HCs), with patients with temporal lobe epilepsy (TLE) showing significantly lower indices compared to HCs. **(C)** Schematic diagram depicting the distribution of regional differences throughout the whole brain, highlighting significant differences between groups. Dark blue: Cluster 1; Light blue: Cluster 2; Green: Cluster 3, Yellow: Cluster 4, Red: Cluster 5.

**Figure 4:**
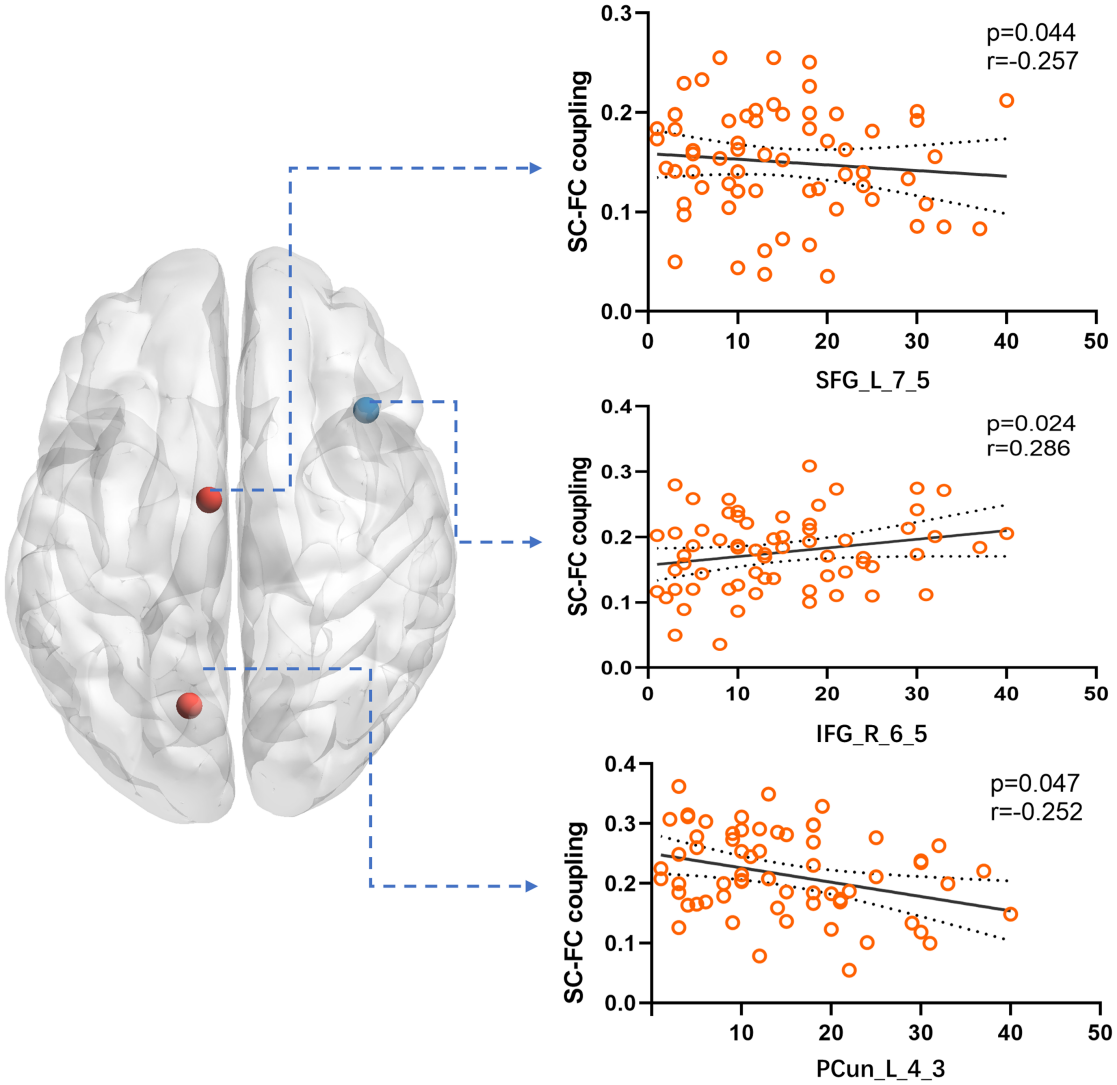
We compared the correlation between the SC–FC coupling strength of nodes in Cluster 5 and various clinical variables. The results showed a significant correlation with the year of seizure duration. Specifically, SFG_L_7_5 and PCun_L_4_3 exhibited a negative correlation, while IFG_R_6_5 showed a positive correlation.

By comparing average cluster SC–FC coupling in each cluster of the three groups, we identified marked discrepancies within the three cohorts in Cluster 3 (*p* = 0.001), Cluster 4 (*p* < 0.001), and Cluster 5 (*p* < 0.001) (one-way ANOVA) (Figure 3A). Post-hoc analysis revealed that the SC–FC coupling strengths in LTLE and RTLE were significantly lower than that in HCs in Cluster 3 (*P*_L_ = 0.001/*P*_R_ = 0.003), Cluster 4 (*P*_L_ = 0.001/*P*_R_ < 0.001), and Cluster 5 (*P*_L_ < 0.001/*P*_R_ < 0.001). In LTLE, the nodes with significantly reduced average intensity of regional SC–FC coupling were mainly located in Cluster 2, whereas they were mainly located in Cluster 5 in RTLE. The within-cluster SC–FC coupling in each cluster was calculated and compared between the LTLE, RTLE, and HC groups using a two-sample *t*-test. The results revealed that the within-cluster SC–FC coupling in Cluster 5 of LTLE and RTLE was significantly lower than that in the HC group (*P*_L_ = 0.0001, *P*_R_ = 0.0005, Tables 2, 3 and Figures 2, 3). Further analysis revealed the relationship between Cluster 5 of LTLE and RTLE and the relevant clinical variables. There was a negative correlation between the year of seizure duration and SFG_L_7_5 and PCun_L_4_3, while a positive correlation was observed with IFG_R_6_5 in Cluster 5 (Figure 4). Through ANCOVA and subsequent *post hoc* tests, we found no significant differences between the LTLE and RTLE.

**Table 2:** The average intensity of SC–FC coupling of nodes in each cluster.

	HC	LTLE	RTLE	*P*-anove	*p*-value		
					RTLE vs. HC	LTLE vs. HC	RTLE vs. LTLE
Cluster 1	0.103 ± 0.019	0.090 ± 0.032	0.082 ± 0.045	0.079	0.027	0.149	0.428
Cluster 2	0.150 ± 0.012	0.141 ± 0.027	0.141 ± 0.023	0.025	0.023	0.015	0.880
Cluster 3	0.176 ± 0.010	0.163 ± 0.025	0.165 ± 0.021	0.001	0.003	0.001	0.605
Cluster 4	0.201 ± 0.011	0.191 ± 0.017	0.190 ± 0.018	<0.001	<0.001	0.001	0.763
Cluster 5	0.247 ± 0.016	0.228 ± 0.021	0.219 ± 0.020	<0.001	<0.001	<0.001	0.079

HC, healthy control; LTLE, left temporary lope epilepsy; RTLE, right temporary lope epilepsy.

**Table 3:** Average intensities of regional SC–FC coupling in each cluster.

	HC	LTLE	RTLE	*p*-value	
				RTLE vs. HC	LTLE vs. HC
Cluster 1	0.195 ± 0.059	0.186 ± 0.072	0.179 ± 0.069	0.528	0.278
Cluster 2	0.146 ± 0.030	0.151 ± 0.024	0.144 ± 0.021	0.408	0.774
Cluster 3	0.191 ± 0.035	0.178 ± 0.035	0.180 ± 0.032	0.101	0.169
Cluster 4	0.254 ± 0.027	0.239 ± 0.022	0.246 ± 0.029	0.014	0.259
Cluster 5	0.518 ± 0.049	0.465 ± 0.073	0.471 ± 0.067	<0.001	<0.001

HC: healthy control; LTLE, left temporary lope epilepsy; RTLE, right temporary lope epilepsy.

## 4. Discussion

Within this study, we determined the intensity of association between the SC and FC profiles of the whole brain and sorted all nodes based on SC–FC coupling status using an automatic clustering algorithm. We discovered that the strength of SC–FC coupling was highest in the visual network and lowest in the limbic system in HCs. Decoupling was observed at those originally highly coupled regions and modules in TLE. The ASFC in Clusters 3–5 of the TLE was significantly less than that of the HCs. In addition, the CSFC in Cluster 5 was significantly lower in patients with TLE. The current study is the first to classifies the whole brain region based on the node SC–FC coupling status and further analyze the discrepancies in the SC–FC coupling intensity in different cluster between HCs and patients with TLE (Hinds et al., 2023).

### 4.1. SC–FC coupling in HC

Various studies are still exploring the correlation between structural and functional connections of neural connection networks. Experts have depicted the human brain as an interactive network of graphics and interactive segments using graph theory and current noninvasive imaging techniques. Connectomics has been introduced to describe brain structures and functional connections. An increasing number of analyses are showing that SC and FC demonstrate a tight and intricate relationship. and some reports have directly proven that the two patterns in the brain are related (Skudlarski et al., 2008; Honey et al., 2009; Wang Z. J. et al., 2015). There are various methods for defining network modules. However, most of the current algorithms are based on functional connectivity (ICA) or brain network topology (Newman’s modularity), but all of the above methods can only be based on a single modality of the network for modular analysis, and cannot efficiently cluster nodes in terms of SC–FC coupling. In our research, we used *k*-means clustering analysis to automatically segment the brain networks. We found that the limbic system was mainly located in Clusters 1 and 2, in which SC–FC coupling was the weakest, and the visual network was in Clusters 4 and 5, with the strongest coupling index. The rich-club system (Collin et al., 2014) and a division of the core structure (Hagmann et al., 2008) are associated with tight integration between SC and FC. Based on myelination and junction patterns of white matter, there can be an anatomical hierarchy reflecting specific functions (Barbas and Rempel-Clower, 1997; Margulies et al., 2016; Burt et al., 2018). Functional activation patterns in visual networks with a higher structural nodal degree and high cortical myelination demonstrate high consistency with their white-matter connectivity profiles. Due to MR imaging artifacts, a limbic system with a lower signal-to-noise ratio may result in weaker SC and FC node degrees and SC–FC coupling (Marquis et al., 2019). In the sensorimotor network, we found that the SC–FC coupling of the prefrontal lobe and paracentric gyrus is also higher, which is consistent with previously identified high coupling near the central sulcus (Koch et al., 2002). Interestingly, Horn et al. discovered that the DMN in the human brain demonstrates notably elevated voxel-by-voxel SC–FC correspondences (Horn et al., 2014). This is largely consistent with our findings, and we also found that the orbital and cingulate gyri were highly coupled in the DMN.

### 4.2. SC–FC coupling in TLE

Brain diseases are linked to significant abnormalities in SC–FC coupling (Alstott et al., 2009; Pons et al., 2010; Zhang et al., 2011; van den Heuvel et al., 2013). There are usually changes in the brain network independent of seizure foci in TLE, which is a common brain network disease. TLE structural and functional connections are influenced by different patterns, according to various studies, with disruptions in both SC and FC being commonly observed in individuals with TLE (Chiang et al., 2015; Trimmel et al., 2021), including the DMN, auditory, and language networks (left frontoparietal network). Our study also revealed that patients with TLE also showed obvious decoupling compared to HCs. There are several factors that may contribute to SC–FC decoupling in TLE. However, a significant reduction in DTI connectivity indicates a white matter abnormality. Multiple noninvasive imaging techniques, including fMRI, DTI, have consistently demonstrated impaired structural and functional connectivity in TLE (Liao et al., 2011; Chiang et al., 2014). We divided each patient’s brain network into five modules that were automatically sorted by the status of regional SC–FC coupling in the HCs. We discovered evidence that the coupling intensity in participants with LTLE and RTLE was less than that in the HC group in the highest-coupled cluster. This suggests that the brain regions with high SC–FC coupling are more likely to decouple in TLE. Previous research has consistently demonstrated elevated levels of SC–FC coupling in the cortical core network and a subset of the structural core in HCs (Hagmann et al., 2008; Collin et al., 2014). In individuals with left- and right TLE, the extent of impairment was more pronounced in functional connections than in structural connections (Chiang et al., 2015). Therefore, the fragile core of the brain network may be more vulnerable to damage during the pathological process (Gollo et al., 2018), resulting in impaired functional connections. As such, we speculated that highly coupled brain regions are more likely to be affected by a decline in functional connections during disease progression. In order to further analyze the reasons, we discovered that several regions in Cluster 5, which has the highest coupling, has a certain correlation with the year of seizure duration. The coupling index of SFG_L_7_5 (a part of left superior frontal gyrus) and PCun_L_4_3 (a part of left precuneus) have a negative correlation with the year, which fully validates our previous conclusion. The coupling index of IFG_R_6_5 (a part of right inferior frontal gyrus) in the ventral attention network is positively correlated with the year of seizure duration, which is consistent to some extent with previous studies (Chiang et al., 2015). This may be related to the rapid propagation and high epileptic index of the ventral attention network during the process of epileptic seizures (Guo et al., 2023).

## 5. Limitations

The limitations of our research include the following points. First, the retrospective design may have limited the reliability of the results. Second, deterministic tractography does not accommodate situations involving prolate or isotropic tensors. As a result of assuming one predominant fiber direction per voxel, tracking may also result in incorrect directions when fibers cross or kiss (Shetty et al., 2014). Future research may use other methods, such as probabilistic tractography. Third, the size of the cohort was relatively small, yielding less power to discern statistically significant discrepancies There is likely to be a strong association between the discrepancies reported in this study, which merits confirmation through studies with larger cohorts. Future studies could further verify our results on an individual basis instead of at the collective level.

## 6. Conclusion

The SC and FC are inconsistently coupled across the brain with spatial heterogeneity, and the coupling degree is highest in the visual network and lowest in the limbic system. In the fifth cluster with the highest degree of coupling in HCs, the average SC–FC coupling index of individuals with TLE was notably less than that of HCs, manifesting that brain regions with high coupling may be more delicate and prone to pathological disruption.

## Data availability statement

The raw data supporting the conclusions of this article will be made available by the authors, without undue reservation.

## Ethics statement

The studies involving humans were approved by Ethics Committee and Institutional Review Board of Xiangya Hospital. The studies were conducted in accordance with the local legislation and institutional requirements. The participants provided their written informed consent to participate in this study. Written informed consent was obtained from the individual(s) for the publication of any potentially identifiable images or data included in this article.

## Author contributions

XH: Conceptualization, Writing – original draft, Writing – review & editing. CZ: Conceptualization, Writing – review & editing, Funding acquisition, Methodology, Validation. FX: Data curation, Funding acquisition, Project administration, Writing – review & editing. DG: Data curation, Methodology, Validation, Writing – review & editing. YD: Conceptualization, Formal analysis, Resources, Validation, Writing – review & editing.

## Footnotes

^1^ https://www.fil.ion.ucl.ac.uk/spm/software/spm12/

^2^ https://fsl.fmrib.ox.ac.uk/fsl/fslwiki/
