# Corrigendum: Structural–functional coupling abnormalities in temporal lobe epilepsy

**DOI:** 10.3389/fnins.2025.1570301

**Published:** 2025-02-18

**Authors:** Xiaoting Huang, Yangsa Du, Danni Guo, Fangfang Xie, Chunyao Zhou

**Affiliations:** ^1^Department of Neurology, Xiangya Hospital, Central South University, Changsha, China; ^2^Department of Radiology, Xiangya Hospital, Central South University, Changsha, China; ^3^Department of Neurosurgery, Xiangya Hospital, Central South University, Changsha, China

**Keywords:** functional connectivity, structural connectivity, temporal lobe epilepsy, structural–functional coupling, human brain

In the published article, there was an error in the Funding statement. The funding information “the Fundamental Research Funds for the Central Universities of Central South University” was erroneously excluded from the Funding statement. The correct Funding statement appears below.

